# Hotel-Dieu Du Creusot

**Published:** 1895-12-28

**Authors:** 


### HOTEL-DIEU DU CREUSOT.

The Hôtel-Dieu du Creusot, situated in the Croix-Menée quarter, which was begun in 1889, was only completed on September 15th last, and has therefore been erected and arranged in accordance with all modern requirements and improvements. It consists of a central block and an isolation ward, laundry and mortuary separated from the principal building and from each other by a well laid-out and well-kept garden.

The principal building is two stories high. On the ground-floor are the surgical wards, on the first floor the medical and on the second floor the store-rooms, linen-rooms, &c.; the rooms on this floor could, however, in case of an epidemic, be quickly converted into temporary wards. The number of beds in the hospital (including the isolation ward) is 132; there are two large wards of twenty-four beds each, the rest being distributed among smaller wards containing from two to eight beds. There are also private rooms somewhat better furnished, containing each two beds, so that a relation or friend of the patient can, if so desired, remain with him at the hospital. The hospital is lit throughout by electricity, and, between the beds in the wards and in every room, there is a movable electric lamp in case any patient should require particular attention during the night. Independently of the perforated panes, thorough ventilation is assured by ventilators placed between the windows, and the building is warmed by hot-air pipes extending from a large cylinder which goes from floor to floor in the centre of the hospital. The furniture is everywhere of a simple character. The beds are of iron; there are no carpets, and corners and angles in the walls have everywhere been rounded off. The isolation ward is situated at the far end of the garden, but is in telephonic communication with the central building.

